# Feasibility and acceptability of suicide prevention therapy on acute psychiatric wards: randomised controlled trial

**DOI:** 10.1192/bjo.2018.85

**Published:** 2019-02-05

**Authors:** Gillian Haddock, Daniel Pratt, Patricia A. Gooding, Sarah Peters, Richard Emsley, Emma Evans, James Kelly, Charlotte Huggett, Ailsa Munro, Kamelia Harris, Linda Davies, Yvonne Awenat

**Affiliations:** Professor of Clinical Psychology, Division of Psychology and Mental Health, School of Health Sciences, University of Manchester, Greater Manchester Mental Health NHS Foundation Trust (formerly Manchester Mental Health and Social Care Trust) and Manchester Academic Health Sciences Centre, UK; Senior Lecturer in Clinical Psychology, Division of Psychology and Mental Health, School of Health Sciences, University of Manchester, Greater Manchester Mental Health NHS Foundation Trust (formerly Manchester Mental Health and Social Care Trust) and Manchester Academic Health Sciences Centre, UK; Senior Lecturer in Psychology, Division of Psychology and Mental Health, School of Health Sciences, University of Manchester, Greater Manchester Mental Health NHS Foundation Trust (formerly Manchester Mental Health and Social Care Trust) and Manchester Academic Health Sciences Centre, UK; Senior Lecturer in Psychology, Division of Psychology and Mental Health, School of Health Sciences, University of Manchester and Manchester Academic Health Sciences Centre, UK; Professor of Medical Statistics and Trials Methodology, Division of Population Health, Health Services Research and Primary Care, School of Health Sciences, University of Manchester, Greater Manchester Mental Health NHS Foundation Trust (formerly Manchester Mental Health and Social Care Trust) and Manchester Academic Health Sciences Centre; and Biostatistics and Health Informatics, Institute of Psychiatry, Psychology and Neuroscience, Kings College London, UK; Trial Therapist, Division of Psychology and Mental Health, School of Health Sciences, University of Manchester, Greater Manchester Mental Health NHS Foundation Trust (formerly Manchester Mental Health and Social Care Trust) and Manchester Academic Health Sciences Centre, UK; Clinical Psychologist, Division of Psychology and Mental Health, School of Health Sciences, University of Manchester; Manchester Academic Health Sciences Centre; and Lancashirecare NHS Foundation Trust, UK; Research Assistant, Division of Psychology and Mental Health, School of Health Sciences, University of Manchester, Greater Manchester Mental Health NHS Foundation Trust (formerly Manchester Mental Health and Social Care Trust) and Manchester Academic Health Sciences Centre, UK; Research Assistant, Division of Psychology and Mental Health, School of Health Sciences, University of Manchester, Greater Manchester Mental Health NHS Foundation Trust (formerly Manchester Mental Health and Social Care Trust) and Manchester Academic Health Sciences Centre, UK; Research Assistant, Division of Psychology and Mental Health, School of Health Sciences, University of Manchester and Manchester Academic Health Sciences Centre, UK; Professor of Health Economics, Division of Population Health, Health Services Research and Primary Care, School of Health Sciences, University of Manchester and Manchester Academic Health Sciences Centre, UK; Research Fellow, Division of Psychology and Mental Health, School of Health Sciences, University of Manchester, Greater Manchester Mental Health NHS Foundation Trust (formerly Manchester Mental Health and Social Care Trust) and Manchester Academic Health Sciences Centre, UK

**Keywords:** Suicide, randomized controlled trial, psychological treatment, inpatient treatment

## Abstract

**Background:**

Suicidal behaviour is common in acute psychiatric wards resulting in distress, and burden for patients, carers and society. Although psychological therapies for suicidal behaviour are effective in out-patient settings, there is little research on their effectiveness for in-patients who are suicidal.

**Aims:**

Our primary objective was to determine whether cognitive–behavioural suicide prevention therapy (CBSP) was feasible and acceptable, compared with treatment as usual (TAU) for in-patients who are suicidal. Secondary aims were to assess the impact of CBSP on suicidal thinking, behaviours, functioning, quality of life, service use, cost-effectiveness and psychological factors associated with suicide.

**Method:**

A single-blind pilot randomised controlled trial comparing TAU to TAU plus CBSP in in-patients in acute psychiatric wards who are suicidal (the Inpatient Suicide Intervention and Therapy Evaluation (INSITE) trial, trial registration: ISRCTN17890126). The intervention consisted of TAU plus up to 20 CBSP sessions, over 6 months continuing in the community following discharge. Participants were assessed at baseline and at 6 weeks and 6 months post-baseline.

**Results:**

A total of 51 individuals were randomised (27 to TAU, 24 to TAU plus CBSP) of whom 37 were followed up at 6 months (19 in TAU, 18 in TAU plus CBSP). Engagement, attendance, safety and user feedback indicated that the addition of CBSP to TAU for in-patients who are acutely suicidal was feasible and acceptable while on in-patient wards and following discharge. Economic analysis suggests the intervention could be cost-effective.

**Discussion:**

Psychological therapy can be delivered safely to patients who are suicidal although modifications are required for this setting. Findings indicate a larger, definitive trial should be conducted.

**Declaration of interest:**

The trial was hosted by Greater Manchester Mental health NHS Trust (formerly, Manchester Mental Health and Social Care NHS Trust). The authors are affiliated to the University of Manchester, Greater Manchester Mental Health Foundation Trust, Lancashire Care NHS Foundation trust and the Manchester Academic Health Sciences Centre. Y.A. is a trustee for a North-West England branch of the charity Mind.

Suicide is a serious public health problem and major cause of preventable death. Suicidality (i.e. suicidal thoughts/behaviours) has a substantial social, personal and economic impact, and on health service provision. In the UK, acute psychiatric care accounts for over two-thirds of National Health Service (NHS) costs^1^ with nearly a third of deaths by suicide occurring in mental health patients.^2^ Between 2005 and 2015, there were 13 576 deaths in the UK (27% of suicides in the general population) where the individual had been in contact with mental health services in the 12 months prior to death.^2^ Suicidal behaviour is an ongoing, recurring problem^3^^,^^4^ and hospital readmissions are common.^5^ The first week of admission to a psychiatric ward is high risk, with approximately one-quarter of suicide deaths occurring during this time.^6^ However, the post-discharge period is also high risk. Data from the UK National Confidential Inquiry into Suicide and Homicide by People with Mental Illness (2017) reported that 17% of all patient suicide fatalities occur during the 3 months following discharge from an acute psychiatric ward.^2^ Given this high prevalence in the ward and post hospital discharge period, there is an urgent need to identify treatments that reduce the likelihood of suicidality in these settings.

There is evidence supporting the use of psychological treatments for suicidality^7^^,^^8^ and National Institute for Health and Care Excellence guidelines recommend cognitive–behavioural therapy (CBT) for the treatment of self-harm.^9^ Several treatment approaches have been evaluated.^8^^,^^10^ However, these have not all directly treated the suicidal, cognitive processes thought to underpin suicidality and none have been evaluated within in-patient settings. One approach that does directly target the underlying psychological mechanisms that drive suicidality is cognitive–behavioural prevention for suicidality in psychosis.^11^ Two trials of the approach, one with people with schizophrenia and one with prisoners who are suicidal showed that the treatment was feasible to deliver and effective in reducing suicidal ideation and suicide probability.^8^^,^^12^ Given that there are high levels of suicidality within acute psychiatric wards and post-discharge, it is important to ascertain whether these treatments can be successfully applied in this population. The primary objective of this trial was to explore whether cognitive–behavioural suicide prevention therapy (CBSP) was feasible and acceptable on acute psychiatric wards. However, we also explored secondary outcomes of suicide behaviour and ideation, functioning, quality of life, service use, preliminary cost-effectiveness and other psychological variables associated with suicidality. The trial protocol has been published.^13^

## Method

### Design

INSITE (Inpatient Suicide Intervention and Therapy Evaluation, trial registration: ISRCTN17890126) was a single-blind pilot randomised controlled trial (RCT) with participants on acute psychiatric wards from one NHS trust in the North West of England, UK. Seventy participants were recruited, and 51 of these were randomly allocated to treatment as usual (TAU) alone or to TAU plus CBSP. Participants were followed up at 6 weeks and 6 months.

The trial used a mixed-methods approach following the Medical Research Council's framework for developing complex interventions. This included qualitative evaluations with staff and patients to inform the delivery of the treatment and to garner their views on its implementation (reported elsewhere, see Awenat *et al*^14^^,^^15^) and the RCT evaluating CBSP is reported here.

### Ethics and governance

The INSITE trial was supported by the NIHR Research for Patient Benefit Programme (PB-PG-1111-26026). The study was approved by the NRES Committee North West – Lancaster (13/NW/0504) and was conducted following guidelines of Good Clinical Practice in accordance with the principles of the Declaration of Helsinki. The study was carried out in collaboration with the national UK charity, Samaritans and a local branch affiliated to the national UK charity, Mind. The trial was overseen by an independent trial steering committee and a Service User Reference Group with previous experience of in-patient suicidality who contributed to the design, execution, analysis and dissemination of the trial.

### Inclusion criteria

Participants were included if they were between 18 and 65 years of age, were in-patients on an acute, psychiatric ward, able to provide informed consent, experienced suicidal thoughts or behaviours within the 3 months prior to admission and had sufficient English language capacity. As the intervention was focused on suicidality rather than on any specific diagnostic group, diagnosis was not considered as a necessary inclusion criterion.

### Recruitment and randomisation

Recruitment for the trial was from May 2014 to January 2016. Eligible participants were identified by in-patient ward staff on eight acute psychiatric wards in one large mental health trust in the North West of the UK. The wards served both inner-city and rural areas providing a large diversity in potential participants. Two wards catered for patients with intensive care psychiatric needs. The wards were served by multidisciplinary teams and specialised in caring for adults of working age. The wards delivered assessment and treatment for a diverse range of mental health conditions, and, included those detained under the Mental Health Act as well as voluntarily admitted patients during an acute, psychiatric crisis and did not aim to provide longer-term rehabilitation services. Participants deemed eligible were provided with an information sheet and informed consent was taken at least 24 h later. Following baseline assessment participants were randomly assigned to, either, TAU or TAU plus CBSP. Randomisation was statistician-led and pseudorandom, carried out using Sealed Envelope software (https://www.sealedenvelope.com/) with group stratification by gender and history of self-harm. Allocation concealment was ensured as participant randomisation codes were not revealed until the participant was recruited into the trial.

Participants were followed up by research assistants masked to treatment allocation at 6 weeks and 6 months following baseline assessment. Procedures for maintaining masking were used. Participants were reminded by research assistants not to disclose their treatment allocation. Where breaches of masking occurred prior to follow-up appointments (18 breaks in masking were recorded during the trial), subsequent data collection was allocated to an alternative research assistant to ensure all data were collected by masked assessors.

### Intervention

The intervention consisted of TAU plus up to 20 CBSP sessions of up to 1 h duration, over 6 months. Participants who were discharged continued their sessions in the community. TAU comprised usual nursing and medical care during the in-patient stay, which included medication, assessments, reviews and evaluation by the ward team. Following discharge, TAU was overseen by the appropriate care professional (for example care coordinator, general practitioner, psychiatrist) and usually involved medical and multidisciplinary review and monitoring. The intervention was guided by a detailed treatment protocol^11^ refined for use with in-patients by the authors and pretrial qualitative interviews.

CBSP is a one-to-one psychological therapy that aims to achieve a detailed understanding of an individual's experiences of suicidality and to change the thinking processes involved in the activation, maintenance and elaboration of suicidal thinking and behaviour using cognitive–behavioural approaches. It was carried out by clinical psychologists meeting the British Association of Behavioural and Cognitive Psychotherapies minimum standards for CBT practice. Treatment fidelity was maintained through adherence to a detailed treatment protocol and weekly supervision. Therapy sessions were audio-recorded with participant's permission and rated by the supervisory team using the Cognitive Therapy Scale for Psychosis.^16^

### Primary outcomes

The primary outcome was feasibility and acceptability of the intervention. This included uptake and attendance at therapy sessions (a minimum of ten sessions attended was anticipated to be an acceptable dose of therapy), attrition (we anticipated a 20% attrition rate) and therapeutic alliance (using the Working Alliance Inventory^17^ administered to patient and therapist at session four and end of therapy).

We also considered the occurrence of research related serious adverse events. Assessment of specific adverse events and serious adverse events were identified from the hospital incident reporting system from randomisation to 6 months follow-up. Anticipated adverse events were predefined as self-harm, harm to others or property and absconding without leave. These data were collected from therapists (uptake and attendance, attrition, therapeutic alliance) and from case notes (specific adverse events) throughout the trial. Acceptability of the intervention was also assessed through qualitative interviews and these are reported elsewhere.^14^^,^^15^

### Secondary outcome measures

Secondary outcomes measures included suicidal ideation, psychopathology, functioning, service use and psychological measures of suicide. Measures were completed at baseline, 6 weeks and 6 months (end of treatment).

#### Measures of intensity and duration of suicidal ideation, and suicidal plans and behaviours

Suicidal ideation, suicidal plans and behaviours were as follows.

- The Suicidal Behaviours Questionnaire – revised, a four-item measure used to establish risk of suicide, revised to report on suicidality in the past 3 months.^18^ The scale has good reliability and validity in clinical and non-clinical samples (at baseline only to assess eligibility).
- The Beck Scale for Suicidal Ideation, a 21-item self-report scale evaluating suicidal ideation, planning and intent over the past week^19^ with good reliability and validity.
- The Suicide Probability Scale, an 18-item self-report questionnaire assessing future suicide probability with good internal consistency and test–retest reliability.^20^
- The Beck Hopelessness Scale, a 20-item self-report questionnaire, measuring negative beliefs about the future over 3 domains over a week.^21^ It is widely used in clinical settings with good reliability and validity.
- A review of clinical records. This review was conducted by a member of the research team to identify episodes of suicidal behaviour and related adverse events from case records.

#### Psychopathology

Psychopathology was assessed using the following scales.

- The Positive and Negative Syndrome Scale (PANSS),^22^ an interviewer-based scale assessing positive and negative psychotic symptoms and general psychopathology with good internal reliability and concurrent validity. All research assistants on the trial were trained to conduct PANSS interviews according to a gold-standard measure used in previous trials.^23^ Intraclass correlations between gold-standard PANSS total scores and research assistant's scores were high (between 0.92 and 0.96). The ongoing reliability of all research assistants was monitored throughout the trial.
- The Psychotic Symptoms Rating Scales (PSYRATS),^24^ a well-validated observer-rated assessment of dimensions of hallucinations and delusions with excellent psychometric properties.
- The Calgary Depression Scale (CDS)^25^ is a nine-item observer-rated measure specifically developed for people with severe mental health problems with high interrater reliability and discriminant validity.

#### Functioning

Function was assessed using the Personal and Social Performance Scale.^26^ It is an interviewer-rated scale used to assess functioning over four domains. The scale has good psychometric properties and is sensitive to differences in social functioning.^27^

#### Quality of life

Quality of life was assessed using the World Health Organization Quality of Life-Brief measure. This is 26- item self-report scale with good to excellent psychometric properties.^28^^,^^29^

#### Negative self-appraisals and other psychological variables associated with suicide

These aspects were assessed using the following measures.

- The Defeat Scale,^30^ a 16-item measure assessing defeat, failed struggle and low social rank over the past week.
- The Entrapment Scale,^30^ a 16-item, self-report scale used to assess feelings of being trapped by internal and external events over the past week. The entrapment and defeat measures both have good psychometric properties.
- The Self-Concept Questionnaire,^31^ a self-report measure of seven components of self-esteem with high reliability and good concurrent and discriminant validity.^32^
- Coping in Stressful Situations Scale,^33^ a 48-item measure assessing three types of coping styles with excellent psychometric properties.

#### Health economic measures

The following health economic measures were used.

- The EQ-5D-5L,^34^ which assesses five domains of mobility, self-care, usual activity, pain/distress and anxiety/depression. Health status profiles were converted into utility values using utility tariffs for EuroQoL to estimate quality-adjusted life-year's (QALY).^34^
- The Use of Services Inventory. This is a trial-specific data-capturing interview evaluating service use, supplemented by case-note review used to estimate costs. Service use information was collected from 6 months before to 6 months after participant randomisation.

We also collected data on sleep, and patient and staff perceptions of acute in-patient wards, which will be reported separately.

### Clinical data analysis

We report all participant flow in accordance with the CONSORT statement.^35^ Demographic data were described using summary statistics. The main efficacy analysis of secondary outcomes was on an intention-to-treat basis with available data from all participants. Pro-rating across scales was used if less than 20% of the items were unobserved, otherwise they were considered as missing. Descriptive statistics were used to summarise quantitative primary outcome data. The secondary outcome measures were analysed using a linear regression model, with baseline measurement of outcome, treatment assignment and site as covariates, at each assessment point separately. The coefficient of the treatment assignment is an estimate of the between-group treatment effect, and can inform potential effect sizes for a future definitive trial. Point estimates and associated 95% confidence intervals are reported rather than tests of statistical significance (*P*-values).

#### Cost-effectiveness analysis

The costs were estimated by multiplying each item of health and social care service use by its unit cost. The unit costs for each type of service used were derived from national databases (price year 2015–16).^36^^–^^38^ The main measure of health benefit was the QALY. Utility values were estimated from the EQ-5D-5L at baseline and follow-up, using published tariffs;^34^ QALYs were estimated from survival and health state utilities. The net costs and QALYs of the CBSP intervention, compared with TAU, were estimated from the perspective of the NHS and social care (costs) and patients (QALYs), for a 6-month time horizon. The primary economic outcome was the incremental cost per QALY gained by the CBSP intervention.

Multiple imputation (predictive mean matching, chained equations, 10 imputations) imputed missing data. Descriptive statistics summarised the total costs and QALYs associated with usual care and the CBSP intervention. Regression models estimated the net costs (GLM – general linear model, gamma, log) and QALYS (OLS – ordinary least squares) of CBSP. The regression included participant demographic characteristics, baseline costs and the EQ-5D visual analogue scale as well as baseline clinical status. The net cost and QALY estimates from the regression analyses were bootstrapped to simulate 10 000 pairs of net cost and net outcomes. These simulations explored the probability that CBSP may be cost-effective compared with usual care alone. In line with this approach, no statistical tests of differences in mean costs or outcomes were conducted, although 95% confidence intervals around the differences are presented.

## Results

### Participants

**Fig. 1:**
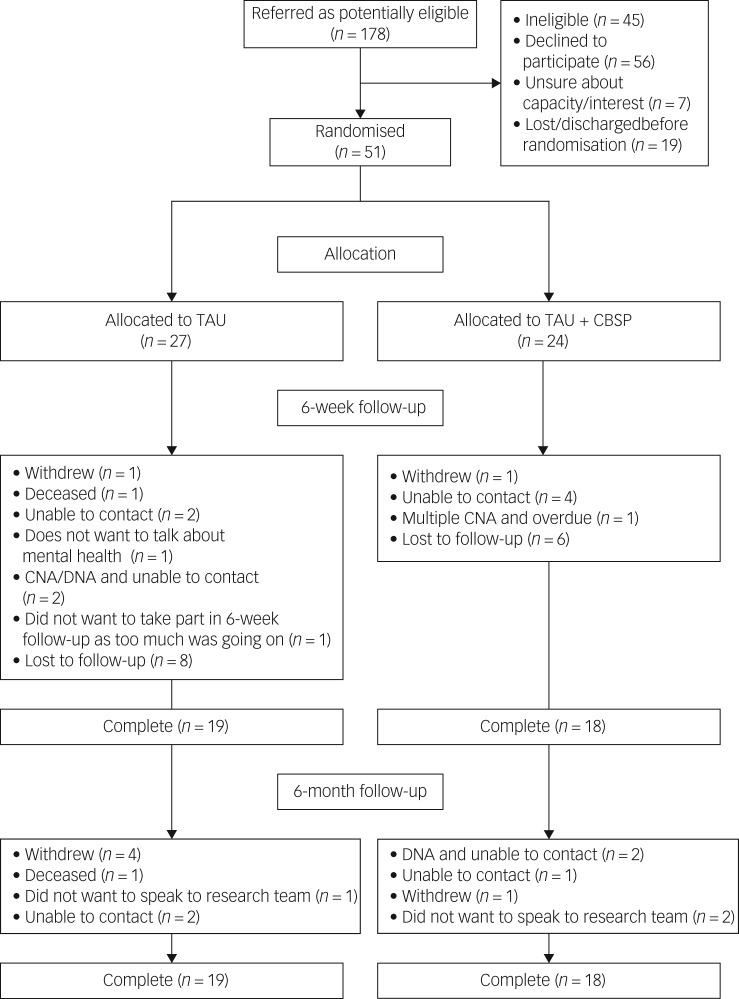
CONSORT diagram to demonstrate participant flow throughout the trial.

TAU, treatment as usual; CBSP, Cognitive Behavioural Suicide Prevention therapy; CNA, could not attend; DNA, did not attend.

In total, 178 potentially eligible patients were referred. Of those, 127 were identified as ineligible, declined to participate, lacked capacity/interest or were lost before randomisation (Fig. 1). There were 70 individuals who gave consent to take part although only 51 participants were randomised because some were discharged before baseline assessment could commence, or these individuals withdrew following consent but before randomisation. Demographic and diagnostic data are presented in Table 1. There was a range of psychiatric diagnoses and/or clinical presentations recorded in case notes with many people receiving multiple diagnoses.

**Table 1:** Demographic characteristics of the participants

Characteristic	TAU (*n* = 27)	CBSP + TAU (*n* = 24)
Gender, *n* (%)		
Women	15 (56)	14 (58)
Men	12 (44)	10 (42)
Legal status, *n* (%)		
Informal	15 (56)	14 (58)
Section 2	6 (22)	5 (21)
Section 3	6 (22)	5 (21)
Ethnicity, *n* (%)		
White British	23 (85)	21 (88)
White other	2 (7)	1 (4)
Black British	1 (4)	1 (4)
Black other	0	1 (4)
Mixed race	1 (4)	0
Marital status, *n* (%)		
Single	21 (78)	17 (71)
Married/relationship	4 (15)	4 (17)
Divorced/widowed	2 (7)	3 (13)
Employment, *n* (%)		
Unemployed	18 (67)	22 (92)
Volunteering	0	1 (4)
Full-time paid employment	1 (4)	0
Sick leave (from work or education)	8 (29)	1 (4)
Case-note recorded diagnosis,^a^ *n* (%)		
Schizophrenia spectrum	15 (56)	10 (42)
Depression	6 (11.8)	8 (15.7)
Anxiety disorder	1 (2)	2 (3.9)
Post-traumatic stress disorder	1 (2)	0
Obsessive–compulsive disorder	1 (2)	1 (2)
Eating disorder	1 (2)	1 (2)
Substance misuse	4 (7.8)	3 (5.9)
Adjustment disorder	1 (2)	0
Attention-deficit hyperactivity disorder	0	1 (2)
Emotionally unstable personality disorder	6 (22)	11 (46)
Unspecified personality disorder	21 (78)	13 (54)
Age, mean (s.d.)	37.04 (12.41)	33.88 (12.18)
Number of admissions,^b^ median (IQR)	1.5 (2)	2 (3)

TAU, treatment as usual; CBSP, Cognitive Behavioural Suicide Prevention therapy; IQR, interquartile range.

a. There was some overlap between diagnosis.

b. Total *n* for data on admissions was 26 for TAU and 21 for the CBSP + TAU group.

### Therapy attendance and uptake

Data on therapy uptake and attendance are shown in Table 2. The vast majority of participants were first seen on in-patient wards and finished therapy in the community. A mean of 6.24 sessions (s.d. = 6.75) were delivered in the in-patient setting with an average of 8.18 sessions (s.d. = 6.53) delivered in the out-patient setting (range 0–20 sessions).

**Table 2:** Therapy uptake

	Mean (s.d.)	Range
Total number of therapy sessions attended	11.35 (6.46)	0–20
Total number of therapy sessions cancelled	5.21 (3.65)	0–14
Total number of therapy sessions not attended	1.83 (2.65)	0–9
Average therapy session length (minutes)	52.05 (8.93)	32–70
Total appointments arranged	18.35 (6.36)	0–25

The average number of sessions attended was just over half of those offered, which was above the ten sessions anticipated to be acceptable. As can be seen from the range, this varied greatly, although 62% achieved a minimum of ten sessions and 86% achieved at least five sessions. Therapy attrition rates were difficult to ascertain because of this variability; however, non-attendance rates were low with an average of 1.83 sessions (range 0–9 sessions) not attended without reason.

Therapeutic alliance (as measured by the WAI), was collected at session 4 and at the end of therapy. However, the completion rate at end of therapy for patients was poor so only data from session 4 is reported here. The therapeutic alliance was similar to that achieved in other psychological intervention trials with complex clients. As is typical, patients rated the therapeutic alliance slightly higher than the therapist (mean  67.16 (s.d. = 10.02) *v.* mean 62.41 (s.d. = 10.20), *t*(37) = 1.46, *P* = .077). Qualitative interviews indicated that both staff and patients viewed the intervention as welcome and acceptable in the form in which it was delivered. See Awenat *et al*^14^^,^^15^ for a detailed report of these findings.

### Serious adverse events

As expected, serious events such as self-harm were common with a total of 255 serious adverse events recorded during the 6-month period of treatment for participants. None were deemed to be research related. There was a large range within participants but no significant differences in the number of these events between TAU and CBSP plus TAU, which totalled 150 (median 1, range 105) and 105 (median 2, range 22), respectively.

### Secondary outcome measures

#### Descriptive statistics

Descriptive data at baseline, 6 week and 6 months for all secondary outcomes are shown in Table 3, and supplementary Tables 1 and 2 available at https://doi.org/10.1192/bjo.2018.85.

**Table 3:** Baseline secondary outcomes

Outcomes	TAU, mean (s.d.); *n*	CBSP + TAU, mean (s.d.); *n*	Total, mean (s.d.); *n*
	(*n* = 27)	(*n* = 24)	(*n* = 51)
Suicide Probability Scale (SPS)			
SPS hopelessness	32.19 (7.76); 27	33 (7.62); 24	32.57 (7.63); 51
SPS ideation	29.26 (8.02); 27	28.5 (8.99); 24	28.9 (8.41); 51
SPS negative evaluation	20.86 (4.13); 27	21.79 (3.43); 24	21.3 (3.8); 51
SPS hostility	17.56 (5.68); 27	17.67 (5.83); 24	17.61 (5.69); 51
SPS total	99.86 (19.83); 27	100.96 (21); 24	100.38 (20.19); 51
SPS probability	84.41 (19.4); 27	83.46 (19.75); 24	83.96 (19.37); 51
SPS suicide risk	3.7 (0.72); 27	3.67 (0.64); 24	3.69 (0.68); 51
Beck Scale for Suicidal Ideation Severity			
Suicidal	21.03 (9.31); 21	23.6 (8.02); 20	22.3 (8.69); 41
Non-suicidal	0.4 (0.89); 5	0.25 (0.5); 4	0.3 (0.71); 9
Missing	1	0	1
Beck Hopelessness Scale total	13.96 (5.82); 26	14.46 (5.64); 24	14.2 (5.68); 50
RSCQ (self concept questionnaire) total	81.88 (31.08); 26	72.91 (24.85); 23	77.67 (28.4); 49
Defeat scale total	50.42 (11.08); 26	51.21 (8.73); 24	50.8 (9.93); 50
Entrapment scale			
External Entrapment scale	26.19 (9.52); 26	26.33 (6.2); 24	26.26 (8.02); 50
Internal Entrapment scale	18.31 (6.4); 26	20.96 (3.21); 24	19.58 (5.25); 50
Entrapment total scale	44.5 (15.23); 26	47.29 (8.37); 24	45.84 (12.38); 50
World Health Organization Quality of Life – Brief measure (WHOQOL)			
WHOQOL Item1	2.5 (1.1); 26	2.21 (0.83); 24	2.36 (0.98); 50
WHOQOL Item2	2.38 (1.2); 26	2 (0.78); 24	2.2 (1.03); 50
WHOQOL Dom1	11.19 (2.8); 26	10.29 (2.45); 24	10.76 (2.65); 50
WHOQOL Dom2	8.05 (3.64); 26	6.86 (2.22); 24	7.48 (3.07); 50
WHOQOL Dom3	9.97 (4.81); 26	10.2 (4.47); 23	10.08 (4.61); 49
WHOQOL Dom4	11.35 (1.91); 26	10.38 (2.74); 24	10.88 (2.37); 50
SCI (sleep condition indicator) total	9.77 (7.39); 26	7.43 (5.65); 23	8.67 (6.67); 49
Coping in Stressful Situations Scale (CISS)			
CISS1 task	43.57 (12.76); 21	37.6 (12.16); 15	41.08 (12.69); 36
CISS2 emotion	60.48 (8.77); 21	57.4 (9.89); 15	59.19 (9.25); 36
CISS3 avoidance	41.43 (13.67); 21	37.73 (11.5); 15	39.89 (12.77); 36
CISS4 distraction	21 (6.91); 21	19.53 (7.43); 15	20.39 (7.06); 36
CISS5 diversion	13.19 (5.71); 21	11.53 (4.39); 15	12.5 (5.2); 36
Personal and Social Performance Scale (PSP)			
PSP activities	4.22 (0.7); 27	4.21 (0.88); 24	4.22 (0.78); 51
PSP relationships	3.63 (0.84); 27	3.63 (1.01); 24	3.63 (0.92); 51
PSP self-care	2.37 (1.31); 27	3.33 (1.55); 24	2.82 (1.49); 51
PSP aggression	2.11 (1.09); 27	2.38 (1.35); 24	2.24 (1.21); 51
PSP total	43.26 (11.27); 27	39.08 (10.91); 24	41.29 (11.19); 51
Psychotic Symptoms Rating Scale (PSYRATS)			
PSYRATS hallucination	13.87 (15.86); 23	12.41 (15.85); 17	13.25 (15.67); 40
PSYRATS delusions	9.62 (9.11); 21	10.06 (9.31); 17	9.82 (9.08); 38
Positive and Negative Syndrome Scale (PANSS)			
PANSS total	70.63 (15.97); 27	76.08 (17.04); 24	73.2 (16.55); 51
Positive PANSS	15.81 (5.7); 27	16.71 (5.93); 24	16.24 (5.77); 51
Negative PANSS	16.19 (6.31); 27	18.58 (6.53); 24	17.31 (6.47); 51
General PANSS	38.74 (7.78); 27	40.83 (8.17); 24	39.73 (7.96); 51
Calgary Depression Scale total	14.22 (5.59); 27	15.08 (4.81); 24	14.63 (5.2); 51

TAU, treatment as usual; CBSP, Cognitive Behavioural Suicide Prevention therapy.

#### Outcomes

No significant differences were observed between the TAU group and TAU plus CBSP group on any secondary outcome measures, across all assessment time points. However, it is noteworthy that raw mean and standard deviation scores for the TAU plus CBSP group show improvements in suicidal ideation, suicide probability, functioning, quality of life, some symptoms of psychosis and depression.

### Cost-effectiveness analysis

Overall 57% (29/51) of participants had complete costs and QALY data (CBSP *n* = 12/24; TAU *n* = 17/27). Supplementary Table 3 summarises utility and costs at baseline and 6-month follow-up. Supplementary Table 4 summarises costs and utility for the multiple imputation data-set. Using the imputed data, the average cost for the TAU group was £35 925 (standard error (s.e.) = £6390, 95% CI £23 050–48 800). This appears higher than the costs of the TAU plus CBSP group (mean £28 566 (s.e. = £6027, 95% CI £16 400–40 733). The average QALY was 0.48 (s.e. = 0.03, 95% CI 0.43–0.54) for the TAU participants, compared with 0.46 (s.e. = 0.03, 95% CI 0.40–0.52) for the people in the TAU plus CBSP group. However, the 95% confidence intervals of the two groups overlap, suggesting no difference in either costs or QALYs.

After taking baseline demographic and clinical characteristics into account and adjusting for the non-normal distribution of costs, the bootstrapped analysis indicates that TAU plus CBSP was associated with a net saving of £15 201 (s.e. = £12 305, 95% CI −£39 318; £8915) and net loss in QALYs of 0.04 (s.e. = 0.03, 95% CI −0.10 to 0.03). The 95% confidence intervals cross zero, suggesting these differences are not statistically significant. Despite the high level of variance in the cost and the QALY, the cost-effectiveness acceptability analysis suggested that TAU plus CBSP may be cost-effective. If policymakers are prepared to accept a net saving of £30 000 for a loss of 1 QALY, then the probability TAU plus CBSP is cost-effective may be around 90%. The net benefit statistic indicates a net benefit of £18 002 (s.d. = £12 632, 2.5th and 97.5th percentiles −£7044 and £42 402, respectively) but the 2.5th and 97.5th percentiles demonstrate uncertainty about the overall cost-effectiveness of TAU plus CBSP.

## Discussion

### The issue of suicidal behaviour on acute wards

Acute psychiatric wards are known to be complex and challenging environments for staff and patients. Recent reports from the Royal College of Psychiatrists,^39^ the Care Quality Commission^40^ and the Schizophrenia Commission^41^ highlight low qualified staff/patient ratios, overreliance on agency workers where staffing is poor, lack of skills in care and insufficient clinical supervision and training. Qualitative research with staff delivering care for suicidal patients^14^ also reflects these issues, reporting that, witnessing suicide fatalities, as well as, repeated suicidal behaviours in in-patient settings is a frequent occurrence. The experience can have a persistent and significant negative impact on staff as well as on the individual patient and their carers. A study with acute, psychiatric in-patient staff identified that 49% were emotionally exhausted, with 29% showing significant psychological distress.^42^ Current ward procedures that are aimed at minimising the occurrence of suicidal behaviours on in-patient wards, such as risk assessments and one-to-one monitoring of adverse behaviours can result in a few opportunities for staff to engage patients in psychosocial interventions.

### Main findings

This preliminary study, however, shows that evidenced-based psychological interventions can be implemented successfully in acute in-patient settings with patients who are perhaps the most distressed and challenging of all mental health patients, providing the opportunity for new treatment options that staff can employ. The trial showed that the intervention and the trial could be delivered with limited harm i.e. no adverse events were found to be trial related suggesting that the methods employed in this trial are feasible for the future roll-out of a large-scale RCT.

### Challenges of delivering the intervention

Nevertheless, there are challenges to delivering the intervention. The take-up of therapy was extremely variable. Not everyone wanted therapy (just over half of those eligible agreed to take part), hence the sample may represent only those most motivated to engage in therapy. Also, of those who did take part, not everyone was able to engage across multiple sessions. However, such participants were in the minority, and significant numbers had a substantial dose of therapy despite severe thoughts of suicidality and exhibiting extreme suicidal behaviours while in hospital. It is notable, that even within these severely distressed participants, it was possible to develop a meaningful therapeutic alliance comparable with that demonstrated with other studies with people with severe mental health problems. It was also notable that a substantial number of the sessions could be carried out in the in-patient phase, rather than following discharge. The therapeutic alliance has been shown to be extremely important in engaging individuals in therapy in other studies, and, crucially, it has been demonstrated to consistently predict long-term outcomes.^43^ Further understanding of staff and patients' views and experiences of therapy will be crucial to inform a future trial and how this may influence long-term outcomes. In addition, exploration of how this has an impact on staff morale is particularly important.

### Limitations

The study had some limitations at should be taken into consideration when interpreting the findings. The study was relatively small and restricted to one, large mental health trust in the North of England. This may limit the generalisability of the findings. However, recruitment took place over a large geographical area over several in-patient wards. The area represented urban and rural catchment areas, with considerable social and economic challenges. In addition, although the psychological therapists who delivered the intervention were not part of the in-patient ward usual treatment team, it was not possible for people receiving both arms of the intervention to be on different wards. This could have led to some cross contamination of approaches. Related to this, the intervention could not entirely take place in isolation of the other treatments that were being delivered on the ward, because therapist contact with ward staff was essential to communicate risk and to support ward staff in facilitating the delivery of the intervention. This may have led to contamination of treatment conditions through staff adopting interventions with other patients who were in the TAU arm. Although this study attempted to minimise this, future studies should ensure that fidelity to the treatment is formally assessed.

The cost-effectiveness results show high levels of variance and uncertainty, likely because of the low sample size and the low numbers of people with complete cost and QALY data. Using the mean cost and QALY data above and assuming a shared standard distribution (QALYs: 0.13; costs: £32 015), initial sample size estimates for a simple RCT design suggest that at the 5% significance level and 80% power, 598 participants would be required to detect a statistically significant difference in costs. For QALYs, this sample doubles to 1310. Further work is needed to explore whether the utility values and estimated QALYs are likely to discriminate between groups, and, how this might relate to other outcomes for example ward environment and staff well-being.

### Implications

Despite these limitations, the study has clear strengths, such as a well-defined protocol, masked outcome assessment, rigorous statistical and cost-effectiveness analyses and reliable assessors of outcome. Findings suggest that psychological interventions should be rigorously evaluated in in-patient settings as they offer a way forward in improving the care of patients who are suicidal. The methods used in this study would be appropriate for a larger, definitive trial.

## Supplementary material

For supplementary material accompanying this paper visit http://dx.doi.org/10.1192/bjo.2018.85. click here to view supplementary material
