# Supplementary material for: Feasibility and acceptability of suicide prevention therapy on acute psychiatric wards: randomised controlled trial
Source: BJPsych Open. 2019 Feb 5;5(1):e14. doi: 10.1192/bjo.2018.85 (PMC6381415; doi:10.1192/bjo.2018.85)
Supplement: Supplementary file 1 [file S2056472418000856sup001.docx]

**Supplementary Table 1: Six week secondary outcome analysis**

| Outcomes | TAU (N=27) | CBSP+TAU (N=24) | Analysis | | |
| --- | --- | --- | --- | --- | --- |
|  | Mean (SD); N | Mean (SD); N | Adjusted mean difference (SE); | 95% CI | Effect size |
| SPS Hopelessness | 30.37 (8.12); 19 | 30.94 (7.04); 17 | -0.90 (2.17) | -5.31, 3.51 | -0.12 |
| SPS Ideation | 25.74 (8.62); 19 | 26.59 (8); 17 | 1.41 (2.12) | -2.90, 5.72 | 0.17 |
| SPS Negative Evaluation | 20.37 (3.5); 19 | 19.96 (3.32); 17 | -0.57 (0.87) | -2.34, 1.20 | -0.15 |
| SPS Hostility | 16.19 (6.13); 19 | 15.84 (5.28); 17 | -0.97 (1.36) | -3.74, 1.80 | -0.17 |
| SPS Total | 92.67 (21.38); 19 | 93.33 (19.38); 17 | -1.51 (4.66) | -10.98, 7.97 | -0.07 |
| SPS Probability | 77.79 (23.74); 19 | 77.35 (19.42); 17 | -0.87 (4.72) | -10.48, 8.74 | -0.04 |
| SPS Suicide risk | 3.42 (0.9); 19 | 3.65 (0.7); 17 | 0.25 (0.20) | -0.16, 0.66 | 0.37 |
| BSS Severity  Suicidal  Non-suicidal  Missing | 15  5  7 | 11  7  6 | Chi square value for non-missing = 0.8458 | p = 0.358 |  |
| BHS Total | 14.14 (6.06); 20 | 14.11 (5.02); 18 | 0.56 (1.40) | -2.29, 3.40 | 0.10 |
| RSCQ Total | 76.92 (25.94); 19 | 74.56 (22.9); 17 | -0.27 (6.31) | -13.12, 12.58 | -0.01 |
| Defeat Total | 47.05 (13.28); 20 | 50 (10.11); 18 | 3.52 (3.53) | -3.65, 10.70 | 0.35 |
| External Entrapment | 21.3 (8.55); 20 | 25.56 (7.87); 18 | 3.57 (2.30) | -1.11, 8.24 | 0.45 |
| Internal Entrapment | 18.35 (6.66); 20 | 20.61 (3.57); 18 | -0.59 (1.22) | -3.08, 1.89 | -0.11 |
| Entrapment total | 39.65 (14.43); 20 | 46.17 (11.03); 18 | 3.41 (3.34) | -3.38, 10.22 | 0.28 |
| WHOQOL Item1 | 2.58 (0.9); 19 | 2.53 (1.01); 17 | -0.04 (0.32) | -0.70, 0.62 | -0.04 |
| WHOQOL Item2 | 2.21 (1.08); 19 | 2.71 (1.26); 17 | 0.56 (0.40) | -0.25, 1.37 | 0.54 |
| WHOQOL Dom1 | 11.48 (2.77); 19 | 11.16 (3.31); 17 | 0.16 (0.81) | -1.50, 1.82 | 0.06 |
| WHOQOL Dom2 | 8.81 (3.26); 19 | 8 (3.34); 17 | -0.35 (0.99) | -2.36, 1.65 | -0.11 |
| WHOQOL Dom3 | 11.51 (3.24); 19 | 11.27 (4.09); 17 | -0.44 (0.93) | -2.34, 1.46 | -0.10 |
| WHOQOL Dom4 | 12.21 (2.06); 19 | 11.76 (3.91); 17 | -0.09 (0.91) | -1.94, 1.76 | -0.04 |
| SCI Total | 10.35 (8.36); 20 | 10.35 (8.99); 17 | 1.91 (2.55) | -3.28, 7.10 | 0.29 |
| CISS1 Task | 41.5 (10.85); 18 | 42.25 (14.01); 12 | 2.93 (4.38) | -6.31, 12.18 | 0.23 |
| CISS2 Emotion | 59 (9.8); 18 | 54.92 (10.54); 12 | -2.78 (3.32) | -9.78, 4.22 | -0.30 |
| CISS3 Avoidance | 41.17 (12.36); 18 | 41.67 (10.85); 12 | 0.30 (4.09) | -8.34, 8.94 | 0.02 |
| CISS4 Distraction | 20.28 (7.35); 18 | 19.42 (5.2); 12 | 0.03 (2.40) | -5.04, 5.09 | 0.00 |
| CISS5 Diversion | 14 (5.93); 18 | 14.75 (5.4); 12 | 0.52 (2.13) | -3.96, 5.01 | 0.10 |
| PSP Activities | 3.85 (1.09); 20 | 3.94 (1.12); 16 | 0.12 (0.37) | -0.63, 0.87 | 0.15 |
| PSP Relationships | 3.4 (0.99); 20 | 3.25 (1); 16 | -0.08 (0.32) | -0.73, 0.57 | -0.09 |
| PSP Self-care | 3.1 (1.37); 20 | 3.19 (1.33); 16 | -0.32 (0.40) | -1.13, 0.50 | -0.21 |
| PSP Aggression | 1.7 (1.22); 20 | 2.75 (1.39); 16 | 0.74 (0.40) | -0.07, 1.55 | 0.61 |
| PSP Total | 46.35 (15.68); 20 | 42.5 (14.62); 16 | -1.57 (4.77) | -11.28, 8.14 | -0.14 |
| PSYRATS Hallucination | 9.06 (10.27); 17 | 8.45 (8.68); 11 | 5.44 (3.50) | -1.83, 12.70 | 0.35 |
| PSYRATS Delusions | 11.88 (16.09); 17 | 7.18 (12.34); 11 | -5.40 (5.88) | -17.68, 6.87 | -0.59 |
| PANSS Total | 66.05 (16.86); 20 | 70.19 (15.61); 16 | 1.70 (4.47) | -7.40, 10.78 | 0.10 |
| Positive PANSS | 13.4 (5.52); 20 | 16.38 (5.34); 16 | 1.97 (1.37) | -0.83, 4.76 | 0.34 |
| Negative PANSS | 16.7 (6.42); 20 | 16.06 (5.43); 16 | -1.49 (1.66) | -4.88, 1.90 | -0.23 |
| General PANSS | 35.95 (8.38); 20 | 37.75 (8.05); 16 | 1.19 (2.30) | -3.49, 5.87 | 0.15 |
| Calgary Total | 12.48 (6.39); 21 | 12.19 (6.74); 16 | -0.65 (1.97) | -4.66, 3.36 | -0.12 |

**Supplementary Table 2:** **Six month secondary outcome analysis**

| Outcomes | TAU (N=27) | CBSP+TAU (N=24) | Analysis | | |
| --- | --- | --- | --- | --- | --- |
|  | Mean (SD); N | Mean (SD); N | Adjusted mean difference (SE) | 95% CI | Effect size |
| SPS Hopelessness | 26.84 (8.69); 19 | 28.88 (6.5); 17 | 1.74 (2.33) | -3.01, 6.50 | 0.23 |
| SPS Ideation | 22 (9.91); 19 | 23.65 (9.17); 17 | 2.26 (2.97) | -3.79, 8.31 | 0.27 |
| SPS Negative Evaluation | 19.21 (3.78); 19 | 19.35 (4.12); 17 | -0.10 (1.13) | -2.41, 2.20 | -0.03 |
| SPS Hostility | 15.53 (4.93); 19 | 15.82 (4.28); 17 | 0.94 (1.37) | -1.84, 3.72 | 0.17 |
| SPS Total | 83.58 (21.37); 19 | 87.7 (20.16); 17 | 5.02 (6.23) | -7.65, 17.70 | 0.25 |
| SPS Probability | 68.32 (24.73); 19 | 71.53 (22.37); 17 | 4.67 (7.11) | -9.80, 19.14 | 0.24 |
| SPS Suicide risk | 3.32 (0.75); 19 | 3.18 (0.81); 17 | -0.12 (0.25) | -0.62, 0.38 | -0.18 |
| BSS Severity  Suicidal  Non-suicidal  Missing | 10  8  9 | 8  7  9 | Chi square value for non-missing = 0.0163 | p = 0.898 |  |
| BHS Total | 12.11 (6.47); 18 | 12.2 (6.38); 15 | 0.42 (2.21) | -4.09, 4.93 | 0.07 |
| RSCQ Total | 86.22 (27.43); 18 | 82.7 (30.13); 15 | -5.48 (8.47) | -22.78, 11.82 | -0.19 |
| Defeat Total | 39.42 (16.32); 19 | 41.93 (11.03); 15 | 3.18 (5.06) | -7.14, 13.50 | 0.32 |
| External Entrapment | 20.56 (10.52); 18 | 19.8 (7.96); 15 | 0.03 (2.92) | -5.95, 6.00 | 0.00 |
| Internal Entrapment | 14.44 (8.71); 18 | 15.87 (6.02); 15 | -0.52 (2.28) | -5.17, 4.14 | -0.10 |
| Entrapment total | 35 (18.35); 18 | 35.67 (13.23); 15 | -0.10 (4.86) | -10.02, 9.82 | -0.01 |
| WHOQOL Item1 | 3.11 (1.02); 18 | 2.67 (0.9); 15 | -0.45 (0.34) | -1.15, 0.26 | -0.46 |
| WHOQOL Item2 | 2.61 (1.14); 18 | 2.2 (1.01); 15 | -0.30 (0.33) | -0.96, 0.37 | -0.29 |
| WHOQOL Dom1 | 11.81 (2.98); 18 | 11.58 (2.96); 15 | 0.15 (0.88) | -1.63, 1.94 | 0.06 |
| WHOQOL Dom2 | 9.15 (2.99); 18 | 8.8 (2.41); 15 | -0.00 (0.83) | -1.70, 1.70 | 0.00 |
| WHOQOL Dom3 | 12.23 (2.72); 17 | 11.02 (4.85); 15 | -1.81 (1.05) | -3.95, 0.34 | -0.39 |
| WHOQOL Dom4 | 12.89 (2.47); 18 | 11.37 (2.98); 15 | -1.43 (0.92) | -3.30, 0.44 | -0.60 |
|  |  |  |  |  |  |
| CISS1 Task | 43.87 (10.13); 15 | 42.64 (15.39); 14 | 0.27 (3.71) | -7.39, 7.92 | 0.02 |
| CISS2 Emotion | 57.07 (9.22); 15 | 54.71 (7.61); 14 | -1.11 (3.06) | -7.42, 5.19 | -0.12 |
| CISS3 Avoidance | 42.93 (12); 15 | 41.29 (11.17); 14 | -1.48 (3.09) | -7.86, 4.89 | -0.12 |
| CISS4 Distraction | 23.87 (6.61); 15 | 22.14 (5.67); 14 | -0.83 (1.99) | -4.95, 3.28 | -0.12 |
| CISS5 Diversion | 12.6 (6.01); 15 | 12.57 (5.83); 14 | -0.46 (1.42) | -3.40, 2.48 | -0.09 |
| PSP Activities | 3.79 (0.63); 19 | 4 (1.03); 16 | 0.16 (0.26) | -0.38, 0.69 | 0.20 |
| PSP Relationships | 3.21 (0.85); 19 | 3.38 (0.89); 16 | 0.19 (0.30) | -0.41, 0.80 | 0.21 |
| PSP Self-care | 2.21 (0.98); 19 | 3.06 (1.39); 16 | 0.54 (0.41) | -0.29, 1.38 | 0.36 |
| PSP Aggression | 2.11 (1.24); 19 | 2.31 (1.25); 16 | 0.20 (0.42) | -0.66, 1.06 | 0.17 |
| PSP Total | 48.26 (11.59); 19 | 46.56 (15.45); 16 | -0.67 (4.56) | -9.96, 8.62 | -0.06 |
| PSYRATS Hallucination | 14.78 (15.78); 18 | 10.56 (14.71); 16 | 1.04 (4.37) | -7.98, 10.06 | 0.07 |
| PSYRATS Delusions | 8.32 (9.16); 19 | 6.63 (8.91); 16 | 0.41 (2.45) | -4.66, 5.48 | 0.05 |
| PANSS Total | 64.41 (18.26); 19 | 63.31 (16.92); 16 | 0.13 (5.58) | -11.24, 11.50 | 0.01 |
| Positive PANSS | 14.37 (6.94); 19 | 12.75 (5.18); 16 | -0.60 (1.51) | -3.68, 2.48 | -0.10 |
| Negative PANSS | 15.16 (6.14); 19 | 15.5 (5.87); 16 | 0.27 (1.65) | -3.08, 3.62 | 0.04 |
| General PANSS | 34.89 (9.19); 19 | 35.06 (9.25); 16 | 0.57 (3.11) | -5.77, 6.91 | 0.07 |
| Calgary Total | 10.32 (6.32); 19 | 10.5 (5.07); 16 | 0.20 (2.01) | -3.90, 4.29 | 0.04 |
| Admissions | 4 (15%) | 5 (21%) |  | Fisher’s exact  p= 0.718 | 0.02 |

**Supplementary Table 3: Utility and costs, by assessment, complete case analysis**

|  | Mean | | Standard Error | | 95% Confidence Interval | |
| --- | --- | --- | --- | --- | --- | --- |
|  | TAU, n=17 | CBSP + TAU, n=12 | TAU, n=17 | CBSP + TAU, n=12 | TAU, n=17 | CBSP + TAU, n=12 |
| Utility | | | | | | |
| Baseline | 0.80 | 0.79 | 0.02 | 0.02 | 0.76;  0.86 | 0.74;  0.83 |
| Follow up, 6 months | 0.82 | 0.77 | 0.02 | 0.03 | 0.78;  0.86 | 0.72;  0.83 |
| Cost: community based services | | | | | | |
| Baseline | £1,668 | £3,310 | £453 | £981 | £740; £2,596 | £1,301; £5,320 |
| Follow up, 6 months | £1,634 | £1,607 | £362 | £396 | £893; £2,376 | £797; £2,417 |
| Cost: primary care | | | | | | |
| Baseline | £10 | £19 | £5 | £8 | <£0;  £21 | £3;  £34 |
| Follow up, 6 months | £8 | £12 | £4 | £7 | -£1;  £16 | -£2;  £26 |
| Cost: accident and emergency care | | |  |  |  |  |
| Baseline | £337 | £533 | £106 | £132 | £119;  £555 | £263;  £803 |
| Follow up, 6 months | £149 | £303 | £68 | £112 | £10;  £288 | £74;  £532 |
| Cost: outpatient care | | | | | | |
| Baseline | £542 | £368 | £209 | £184 | £113;  £970 | -£9;  £746 |
| Follow up, 6 months | £490 | £410 | £230 | £147 | £20; £961 | £108; £711 |
| Cost: inpatient care | | | | | | |
| Baseline | £21,172 | £20,203 | £4,679 | £4,727 | £11,588; £30,756 | £10,521; £29,885 |
| Follow up, 6 months | £39,230 | £26,927 | £8,618 | £8,469 | £21,576; £56,884 | £9,579; £44,274 |
| Cost: CBSP | | | | | | |
| Follow up, 6 months | £0 | £1,260 | £0 | £169 | £0 | £914; £1,606 |

**Supplementary Table 4: Utility and costs, by assessment, multiple imputation analysis**

|  | Mean | | Standard Error | | 95% Confidence Interval | |
| --- | --- | --- | --- | --- | --- | --- |
|  | TAU, n=27 | CBSP + TAU, n=23 | TAU, n=27 | CBSP + TAU, n=23 | TAU, n=27 | CBSP + TAU, n=23 |
| Utility | | | | | | |
| Baseline | 0.81 | 0.79 | 0.02 | 0.01 | 0.77;  0.84 | 0.76;  0.81 |
| Follow up, 6 months | 0.82 | 0.80 | 0.02 | 0.02 | 0.77;  0.86 | 0.76;  0.84 |
| Cost: community based services | | | | | | |
| Baseline | £1,137 | £2,487 | £315 | £570 | £503;  £1,771 | £1,340;  £3,633 |
| Follow up, 6 months | £1,359 | £1,635 | £263 | £294 | £828;  £1,889 | £1,042;  £2,228 |
| Cost: primary care | | | | | | |
| Baseline | £15 | £18 | £5 | £6 | £5;  £25 | £6;  £29 |
| Follow up, 6 months | £14 | £9 | £6 | £4 | £2;  £27 | £1;  £18 |
| Cost: accident and emergency care | | | | | | |
| Baseline | £300 | £572 | £72 | £84 | £156;  £444 | £404;  £740 |
| Follow up, 6 months | £117 | £238 | £47 | £63 | £22;  £212 | £111;  £364 |
| Cost: outpatient care | | | | | | |
| Baseline | £414 | £273 | £143 | £102 | £126;  £701 | £68;  £478 |
| Follow up, 6 months | £353 | £304 | £163 | £86 | £25;  £682 | £132;  £477 |
| Cost: inpatient care | | | | | | |
| Baseline | £20,892 | £25,564 | £3,184 | £4,599 | £14,487;  £27,297; | £16,313;  £34,815 |
| Follow up, 6 months | £34,082 | £25,157 | £6,436 | £5,940 | £21,105;  £47,058 | £13,190;  £37,125 |
| Cost: CBSP | | | | | | |
| Follow up, 6 months | £0 | £1,046 | £0 | £132 | £0 | £780;  £1,313 |

Appendix 1: CONSORT 2010 checklist of information to include when reporting a pilot or feasibility trial

| Section/Topic | Item No | Checklist item | Reported on page No |
| --- | --- | --- | --- |
| Title and abstract | | | |
|  | 1a | Identification as a pilot or feasibility randomised trial in the title | 1 |
|  | 1b | Structured summary of pilot trial design, methods, results, and conclusions (for specific guidance see CONSORT abstract extension for pilot trials) | 2 |
| Introduction | | | |
| Background and objectives | 2a | Scientific background and explanation of rationale for future definitive trial, and reasons for randomised pilot trial | 3-5 |
|  | 2b | Specific objectives or research questions for pilot trial | 5 |
| Methods | | | |
| Trial design | 3a | Description of pilot trial design (such as parallel, factorial) including allocation ratio | 5-6 |
|  | 3b | Important changes to methods after pilot trial commencement (such as eligibility criteria), with reasons | - |
| Participants | 4a | Eligibility criteria for participants | 6-7 |
|  | 4b | Settings and locations where the data were collected | 7 |
|  | 4c | How participants were identified and consented | 7 |
| Interventions | 5 | The interventions for each group with sufficient details to allow replication, including how and when they were actually administered | 7-8 |
| Outcomes | 6a | Completely defined prespecified assessments or measurements to address each pilot trial objective specified in 2b, including how and when they were assessed | 8-11 |
|  | 6b | Any changes to pilot trial assessments or measurements after the pilot trial commenced, with reasons | - |
|  | 6c | If applicable, prespecified criteria used to judge whether, or how, to proceed with future definitive trial | 8 |
| Sample size | 7a | Rationale for numbers in the pilot trial | Feasibility trial only |
|  | 7b | When applicable, explanation of any interim analyses and stopping guidelines | Not applicable |
| Randomisation: |  |  |  |
| Sequence  generation | 8a | Method used to generate the random allocation sequence | 7 |
|  | 8b | Type of randomisation(s); details of any restriction (such as blocking and block size) | 7 |
| Allocation  concealment  mechanism | 9 | Mechanism used to implement the random allocation sequence (such as sequentially numbered containers), describing any steps taken to conceal the sequence until interventions were assigned | 7 |
| Implementation | 10 | Who generated the random allocation sequence, who enrolled participants, and who assigned participants to interventions | 7 |
| Blinding | 11a | If done, who was blinded after assignment to interventions (for example, participants, care providers, those assessing outcomes) and how | 7 |
|  | 11b | If relevant, description of the similarity of interventions | 7-8 |
| Statistical methods | 12 | Methods used to address each pilot trial objective whether qualitative or quantitative | 12-13 |
| Results | | | |
| Participant flow (a diagram is strongly recommended) | 13a | For each group, the numbers of participants who were approached and/or assessed for eligibility, randomly assigned, received intended treatment, and were assessed for each objective | 13 |
|  | 13b | For each group, losses and exclusions after randomisation, together with reasons | 13-14 |
| Recruitment | 14a | Dates defining the periods of recruitment and follow-up | 9 |
|  | 14b | Why the pilot trial ended or was stopped | Trial not stopped |
| Baseline data | 15 | A table showing baseline demographic and clinical characteristics for each group | 26 |
| Numbers analysed | 16 | For each objective, number of participants (denominator) included in each analysis. If relevant, these numbers  should be by randomised group | 29-33 |
| Outcomes and estimation | 17 | For each objective, results including expressions of uncertainty (such as 95% confidence interval) for any  estimates. If relevant, these results should be by randomised group | 29-33 |
| Ancillary analyses | 18 | Results of any other analyses performed that could be used to inform the future definitive trial | 14-15 |
| Harms | 19 | All important harms or unintended effects in each group (for specific guidance see CONSORT for harms) | 15 |
|  | 19a | If relevant, other important unintended consequences | None relevant |
| Discussion | | | |
| Limitations | 20 | Pilot trial limitations, addressing sources of potential bias and remaining uncertainty about feasibility | 17-18 |
| Generalisability | 21 | Generalisability (applicability) of pilot trial methods and findings to future definitive trial and other studies | 19 |
| Interpretation | 22 | Interpretation consistent with pilot trial objectives and findings, balancing potential benefits and harms, and  considering other relevant evidence | 17-19 |
|  | 22a | Implications for progression from pilot to future definitive trial, including any proposed amendments | 19 |
| Other information | | |  |
| Registration | 23 | Registration number for pilot trial and name of trial registry | NIHR ISRCTN17890126 |
| Protocol | 24 | Where the pilot trial protocol can be accessed, if available | https://trialsjournal.biomedcentral.com/articles/10.1186/s13063-016-1192-9 |
| Funding | 25 | Sources of funding and other support (such as supply of drugs), role of funders | NIHR |
|  | 26 | Ethical approval or approval by research review committee, confirmed with reference number | NRES Committee North West – Lancaster 13/NW/0504 |

**Appendix 2: CHEERS checklist -** Items to include when reporting economic evaluations of health interventions

| **Section/item** | **Item No** | **Recommendation** | **Reported on page No/ line No** |
| --- | --- | --- | --- |
| **Title and abstract** | | | |
| Title | 1 | Identify the study as an economic evaluation or use more specific terms such as “cost-effectiveness analysis”, and describe the interventions compared. | Not included since  primary objective is to  assess feasibility and  acceptability of intervention |
| Abstract | 2 | Provide a structured summary of objectives, perspective, setting, methods (including study design and inputs), results (including base case and uncertainty analyses), and conclusions. | Page 2/lines 7-27 |
| **Introduction** | | | |
| Background and objectives | 3 | Provide an explicit statement of the broader context for the study. | Pages 4-5/lines 42-87 |
|  |  | Present the study question and its relevance for health policy or practice decisions. | Page 5/lines 82-87 |
| **Methods** | | | |
| Target population and subgroups | 4 | Describe characteristics of the base case population and subgroups analysed, including why they were chosen. | Population: Page 5/lines 80-82; page 6/lines 92-93; Sample inclusion criteria: page 7/lines 112-116.  No sub-group analysis. |
| Setting and location | 5 | State relevant aspects of the system(s) in which the decision(s) need(s) to be made. | Page 6/lines 92-93 |
| Study perspective | 6 | Describe the perspective of the study and relate this to the costs being evaluated. | Page 12-13/lines 239-240 |
| Comparators | 7 | Describe the interventions or strategies being compared and state why they were chosen. | Interventions Page 6/lines 94-95; Page 8/lines 136-147  Rationale Page 4-5/Lines 62-83 |
| Time horizon | 8 | State the time horizon(s) over which costs and consequences are being evaluated and say why appropriate. | Page 13/line 241 |
| Discount rate | 9 | Report the choice of discount rate(s) used for costs and outcomes and say why appropriate. | No discounting – not relevant for 6 month time-frame |
| Choice of health outcomes | 10 | Describe what outcomes were used as the measure(s) of benefit in the evaluation and their relevance for the type of analysis performed. | Page 11/lines 208 -210; Page 12/lines 236-239 |
| Measurement of effectiveness | 11a | *Single study-based estimates:* Describe fully the design features of the single effectiveness study and why the single study was a sufficient source of clinical effectiveness data. | Pages 6/lines 90-101  Page 7/lines 112-133 |
|  | 11b | *Synthesis-based estimates:* Describe fully the methods used for identification of included studies and synthesis of clinical effectiveness data. | Not applicable |
| Measurement and valuation of preference based outcomes | 12 | If applicable, describe the population and methods used to elicit preferences for outcomes. | Not applicable |
| Estimating resources and costs | 13a | *Single study-based economic evaluation:* Describe approaches used to estimate resource use associated with the alternative interventions. Describe primary or secondary research methods for valuing each resource item in terms of its unit cost. Describe any adjustments made to approximate to opportunity costs. | Page 11/lines 211-214; Page 12/lines 234-236 |
|  | 13b | *Model-based economic evaluation:* Describe approaches and data sources used to estimate resource use associated with model health states. Describe primary or secondary research methods for valuing each resource item in terms of its unit cost. Describe any adjustments made to approximate to opportunity costs. | Not applicable |
| Currency, price date, and conversion | 14 | Report the dates of the estimated resource quantities and unit costs. Describe methods for adjusting estimated unit costs to the year of reported costs if necessary. Describe methods for converting costs into a common currency base and the exchange rate. | Page 7, line 119; page 12/line 236 |
| Choice of model | 15 | Describe and give reasons for the specific type of decision-analytical model used. Providing a figure to show model structure is strongly recommended. | Not applicable |
| Assumptions | 16 | Describe all structural or other assumptions underpinning the decision-analytical model. | Not applicable |
| Analytical methods | 17 | Describe all analytical methods supporting the evaluation. This could include methods for dealing with skewed, missing, or censored data; extrapolation methods; methods for pooling data; approaches to validate or make adjustments (such as half cycle corrections) to a model; and methods for handling population heterogeneity and uncertainty. | Page 13/lines 242-252 |
| **Results** | | | |
| Study parameters | 18 | Report the values, ranges, references, and, if used, probability distributions for all parameters. Report reasons or sources for distributions used to represent uncertainty where appropriate. Providing a table to show the input values is strongly recommended. | Not applicable |
| Incremental costs and outcomes | 19 | For each intervention, report mean values for the main categories of estimated costs and outcomes of interest, as well as mean differences between the comparator groups. If applicable, report incremental cost-effectiveness ratios. | Page 16-17/lines 273-292; Supplementary Tables 3 and 4 |
| Characterising uncertainty | 20a | *Single study-based economic evaluation:* Describe the effects of sampling uncertainty for the estimated incremental cost and incremental effectiveness parameters, together with the impact of methodological assumptions (such as discount rate, study perspective). | Page 16-17/lines 273-292; Supplementary Tables 3 and 4 |
|  | 20b | *Model-based economic evaluation:* Describe the effects on the results of uncertainty for all input parameters, and uncertainty related to the structure of the model and assumptions. | Not applicable |
| Characterising heterogeneity | 21 | If applicable, report differences in costs, outcomes, or cost-effectiveness that can be explained by variations between subgroups of patients with different baseline characteristics or other observed variability in effects that are not reducible by more information. | Not applicable |
| **Discussion** | | | |
| Study findings, limitations, generalisability, and current knowledge | 22 | Summarise key study findings and describe how they support the conclusions reached. Discuss limitations and the generalisability of the findings and how the findings fit with current knowledge. | Page 17/lines 286-292; Page 20/lines 342-349 |
| **Other** | | | |
| Source of funding | 23 | Describe how the study was funded and the role of the funder in the identification, design, conduct, and reporting of the analysis. Describe other non-monetary sources of support. | Page 3, lines 29-31 |
| Conflicts of interest | 24 | Describe any potential for conflict of interest of study contributors in accordance with journal policy. In the absence of a journal policy, we recommend authors comply with International Committee of Medical Journal Editors recommendations. | Page 3/lines 35-37 |

For consistency, the CHEERS statement checklist format is based on the format of the CONSORT statement checklist
